# SynFlow: an interactive online genome structural variant viewer

**DOI:** 10.1093/bioinformatics/btag567

**Published:** 2026-07-29

**Authors:** Marilyne Summo, Gaëtan Droc, Mathieu Rouard, Gautier Sarah

**Affiliations:** UMR AGAP Institut, Univ Montpellier, CIRAD, INRAE, Institut Agro, Montpellier 34398, France; CIRAD, UMR AGAP Institut, Montpellier 34398, France; South Green Bioinformatics Platform, French Institute of Bioinformatics (IFB); Bioversity; CIRAD; INRAE; IRD, Montpellier 34398, France; UMR AGAP Institut, Univ Montpellier, CIRAD, INRAE, Institut Agro, Montpellier 34398, France; CIRAD, UMR AGAP Institut, Montpellier 34398, France; South Green Bioinformatics Platform, French Institute of Bioinformatics (IFB); Bioversity; CIRAD; INRAE; IRD, Montpellier 34398, France; South Green Bioinformatics Platform, French Institute of Bioinformatics (IFB); Bioversity; CIRAD; INRAE; IRD, Montpellier 34398, France; Bioversity International, Parc Scientifique Agropolis II, Montpellier 34397, France; UMR AGAP Institut, Univ Montpellier, CIRAD, INRAE, Institut Agro, Montpellier 34398, France; South Green Bioinformatics Platform, French Institute of Bioinformatics (IFB); Bioversity; CIRAD; INRAE; IRD, Montpellier 34398, France

## Abstract

**Motivation:**

Structural variations (SVs), including inversions, translocations (TRAs), duplications, and large insertions or deletions, are key drivers of genome evolution and phenotypic diversity. With the increasing number of high-quality, chromosome-scale genome assemblies, the ability to detect and interpret SVs has become a crucial aspect of modern genomics. While SV detection has advanced, most visualization methods produce static plots that fall short when researchers, particularly in comparative genomics, need to interactively explore large datasets, zoom into specific genomic regions, or dynamically filter structural events in real time.

**Results:**

To address this gap, we introduce SynFlow, a lightweight, web-based interactive application specifically designed for exploring and visualizing SVs identified by SyRI. We demonstrate that SynFlow can reproduce complex static synteny plots published in literature, but transforms them into dynamic, shareable visualizations that support real-time filtering, reordering, and deep exploration of specific SVs, including TRAs. SynFlow is available as a web server and offers multiple entry points: browsing precomputed datasets (e.g. banana and grapevine genomes), uploading user-provided SyRI outputs, or running an integrated workflow to produce and visualize SVs on the fly.

**Availability and implementation:**

https://synflow.southgreen.fr; source code https://github.com/SouthGreenPlatform/synflow; preprocessing Snakemake workflow https://gitlab.cirad.fr/agap/cluster/snakemake/synflow.

## 1 Introduction

Comparative genomics has entered a phase where long-read sequencing data and telomere-to-telomere assemblies and multi-haplotype references are increasingly available across species, placing structural variation (SV) and synteny more than ever at the centre of functional and evolutionary inference (Liu *et al.* 2024). This has led to the development of tools for detecting and interpreting SV at the assembly level, with tools such as SyRI (Goel *et al.* 2019) and MCScanX (Wang *et al.* 2012), that are widely adopted and highly cited methods for whole genome assembly-based identification of genomic rearrangements.

However, once SVs are identified, researchers also need effective ways to visualize and interpret them in a comparative context. To address this, several visualization solutions have been developed, which fall into two main categories: static or command-line figure generators [e.g. plotsr (Goel and Schneeberger 2022), SVbyEye (Porubsky *et al.* 2025), SYNY (Julian and Pombert 2024), and ntSynt-viz (Coombe *et al.* 2025)] and general-purpose genome browsers [e.g. JBrowse 2 (Diesh *et al.* 2023)].

While the former excel at producing publication-quality static figures and the latter provide flexible interactive environments, in practice, researchers must either install packages and script outputs, configure browsers to achieve only partial interactivity (e.g. they often lack real-time filtering by SV type), or rely on specialized tools that remain limited to static displays. Importantly, very few platforms currently offer a straightforward online server where users can quickly and easily upload or compute outputs from widely used software and obtain interactive, shareable, and filterable visualizations of synteny and SV.

To address this gap, we present SynFlow, available as a web server for both visualization and computation, providing an interactive browser-based environment for exploring SV and synteny across multiple whole-genome assemblies.

## 2 Methods

### 2.1 Client-server architecture

SynFlow is implemented as a web application following a client-server architecture. The front-end handles user interactions and visualization, while the back end executes the analysis pipeline. The interface is built using standard web technologies (HTML, CSS, and JavaScript). Interactive data visualization is powered by D3.js, enabling scalable rendering. Bootstrap ensures a consistent and responsive layout across devices. jQuery is used for simplified event handling and DOM manipulation. On the server side, Node.js manages file uploads, executes the analysis pipeline, monitors progress, and collects the resulting output files.

### 2.2 Data import and supported file formats

SynFlow needs BEDPE files to generate the SV visualization as this file format explicitly captures paired genomic coordinates required for whole-genome comparisons. In SynFlow, the BEDPE is extended with SyRI-derived annotation columns that describe the specific type of structural variant: inversion (INV), translocation (TRA), duplication (DUP), or deletion (DEL). These files contain paired genomic coordinates along with their SV annotations, enabling immediate visualization of synteny blocks and structural rearrangements. BEDPE input is well-suited when users have pre-computed alignments, enabling them to focus directly on comparative visualization and interpretation.

**Figure 1:**
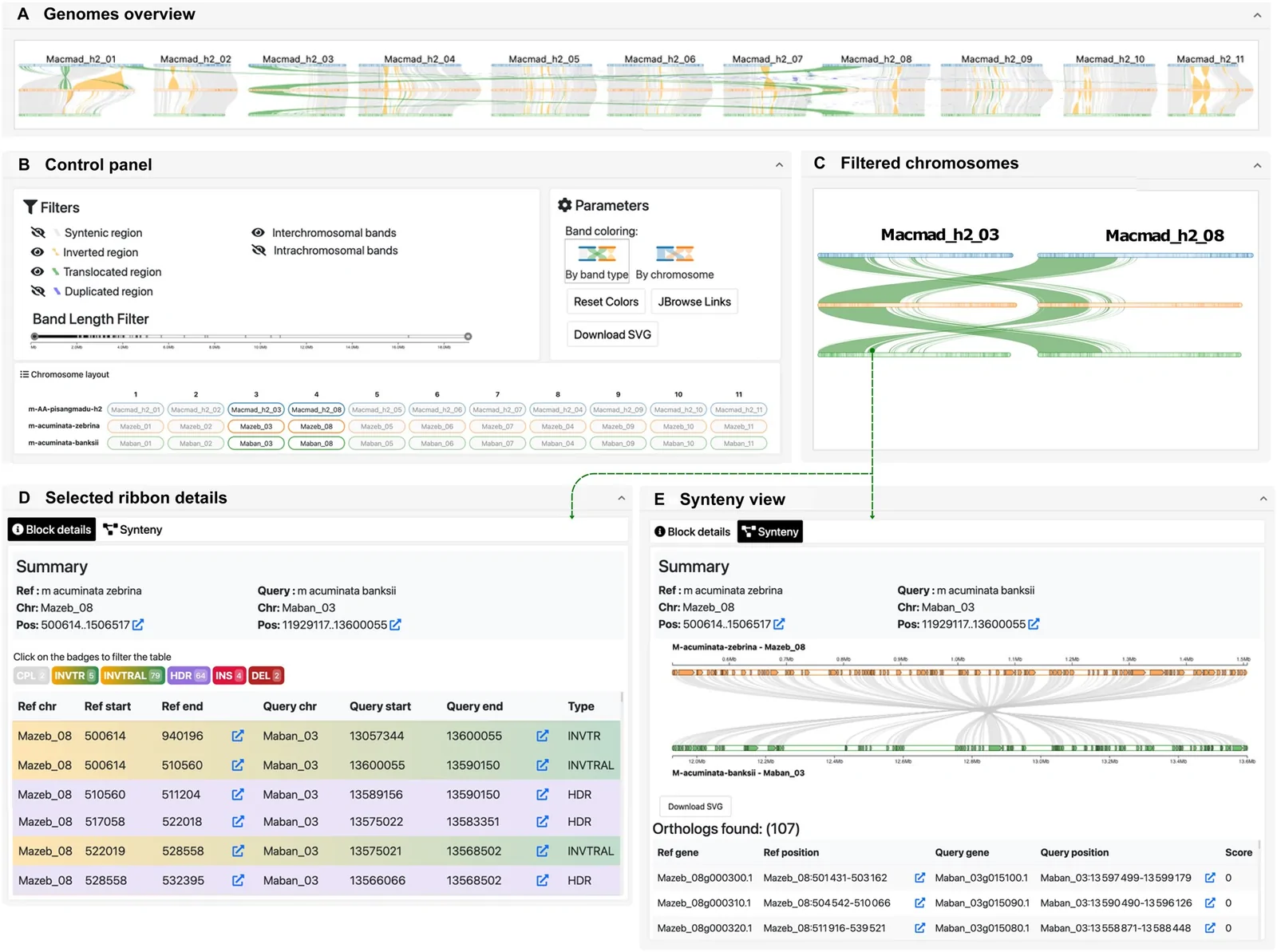
Interactive exploration of structural variation and orthology in SynFlow. (A) Overview of chromosome-scale synteny and structural rearrangements between two *Musa* genomes. Coloured ribbons represent different types of structural variants as detected by SyRI. (B) Control panel allowing users to filter, reorder, or hide chromosomes in real time. (C) Filtered graph showing a detailed, zoomed view of rearrangements between selected chromosomes. (D) Structural variant list with dynamic filtering capabilities of the selected ribbon. (E) Synteny view showing local structural variation and orthology prediction.

Alternatively, users can generate a BEDPE input file by directly providing genome assemblies (FASTA) and their corresponding annotation files (GFF), which are processed through an original workflow built with Snakemake (Köster and Rahmann 2012) (Fig. 1, available as supplementary data at *Bioinformatics* online). This workflow first performs whole-genome alignment [e.g. with Nucmer (Marçais *et al.* 2018) or Minimap2 (Li 2018)], followed by SV detection using SyRI (Goel *et al.* 2019) and synteny detection using MCScanX (Wang *et al.* 2012). SyRI is most suitable when the genomes being compared share the same chromosome number. However, when assemblies differ in chromosome number, like in interspecific analysis, the pipeline automatically switches to MCScanX, a widely used tool for synteny and rearrangement detection under such conditions. The resulting outputs are then formatted for visualization in SynFlow.

## 3 Results

### 3.1 Description of the interface

SynFlow is a web-based application that allows users to explore genome alignments and SVs for eukaryotic organisms, including plants, animals, and fungi. SynFlow provides an overview of all chromosomes and SVs across two or more genomes (Fig. 1A and Fig. 2, available as supplementary data at *Bioinformatics* online). Users can quickly inspect ribbons and coordinates through simple mouseover interactions. The control panel offers a summary of syntenic, inverted, translocated, and duplicated regions, as well as band length distributions. By default, two colouring modes are available, by SV type or by chromosome, allowing users to focus either on evolutionary event types (e.g. fusion or fission) or on variant types. A layout view showing chromosome numbers and naming conventions is also provided to support further interaction.

These interactive features allow users to zoom in and out of chromosomes, reorder or hide them, and filter bands to highlight specific SVs, such as TRAs (Fig. 1B and C). Chromosome and ribbon colours can be customized, and ribbons may be selected individually or in groups based on their type or sequence proximity. The final visualization can be easily exported as scalable vector graphics files, ensuring high-resolution quality that can be easily edited or integrated into manuscripts.

Leveraging SyRI-like outputs, SynFlow distinguishes and displays INVs, TRAs, DUPs, and DELs at their genomic locations. Users can navigate from whole-genome overviews to detailed neighbourhood views in real time. These local views display SV block details alongside their corresponding gene collinearity information, which is based on protein-based orthology predictions (Fig. 1D and E). These orthologous genes are visualized as ribbons between chromosomes and listed in a dynamic table. This table allows users to actively search and filter structural variant entries by coordinates, size, or associated ortholog information, connecting the large-scale SVs with fine-grained gene-level relationships in a single interactive environment.

SynFlow integrates with external genome browsers such as JBrowse, allowing users to click through from synteny panels into detailed track-based contexts. Each entry includes links to external genome browser views, whenever available, enabling users to verify and explore underlying annotations. SynFlow can be configured to connect to multiple JBrowse installations, providing flexible integration across distributed datasets. A feature matrix comparing the functionalities of a panel of SV visualization tools has been added to Table 1, available as supplementary data at *Bioinformatics* online.

### 3.2 ‘Bring-your-own-data’ mode

SynFlow is designed as a ‘bring-your-own-data’ service that supports several modes of input and processing. Users can select existing files already stored on the server, upload their own assemblies or SyRI outputs, or browse remote repositories via URLs. Once files are available, the system can trigger an integrated Snakemake pipeline that performs whole-genome alignment, runs SyRI (or MCScanX when appropriate), and generates the corresponding SV catalogue. This workflow removes the technical burden of installing packages and scripting custom figure generation while ensuring compatibility with widely used comparative genomics pipelines. Notably, unlike SyRI, the pipeline allows comparisons between genomes that differ in chromosome numbers, with the interface supporting their graphical visualization.

Regardless of the upload method used, a genome composition matrix is provided on the interface to facilitate visualization of the number of genomes, the available pairwise comparisons and their anchors (i.e. orthologous genes delineating syntenic blocks), the presence of gene annotation files for ortholog exploration, and whether a configuration for redirection to JBrowse has been provided.

In order to illustrate the figure quality, we reproduced the comparative visualization from the recent study of banana speciation [Fig. 3 in Martin *et al.* (2025)] (Fig. 3, available as supplementary data at *Bioinformatics* online). While the original figure was static, SynFlow renders the same data interactively, allowing users to zoom, filter specific event classes, reorder genomes, and manipulate the view in real time. This demonstrates SynFlow’s ability to transform static illustrations from existing published results into dynamic visualizations, significantly enhancing their value for follow-up analysis.

### 3.3 Deployment and portability

Although SynFlow is available as a public web service, it is also designed with portability in mind. The application is packaged as a single-page web app and can be deployed in a wide range of environments, including institutional servers, cloud object storage, or local machines. For research groups with proprietary or sensitive data, SynFlow is fully containerized and easily deployable via Docker on any local or institutional server, providing a robust and private environment for analysis. Detailed installation instructions and configuration options are provided in the project documentation (https://synflow.readthedocs.io/en/latest), enabling users to adapt the platform to their own infrastructure. Scalability for visualization is contingent on the user’s browser and local machine resources, since all visualizations and computations are handled client-side in JavaScript.

## 4 Conclusion

SynFlow addresses a major bottleneck in comparative genomics: the lack of an easy and interactive way to explore SV across multiple whole-genome assemblies. Unlike existing static visualization tools or command-line tools, SynFlow is the first web-native, SyRI-compatible viewer that requires no coding expertise, supports real-time navigation and filtering, and enables comparative visualization even when genomes differ in chromosome number. Furthermore, it provides maximum flexibility as it can be deployed either as a public service or locally via Docker. Ultimately, SynFlow transforms static comparative genomic figures into dynamic analyses, lowering the barrier for both specialists and non-specialists to investigate complex genome rearrangements.

## Supplementary Material

btag567_Supplementary_Data

## Data Availability

SynFlow is freely available at https://synflow.southgreen.fr. The source code is available at https://github.com/SouthGreenPlatform/synflow. The preprocessing workflow is available at https://gitlab.cirad.fr/agap/cluster/snakemake/synflow.
