# Supplementary material for: SynFlow: an interactive online genome structural variant viewer
Source: Bioinformatics. 2026 Jul 29;42(8):btag567. doi: 10.1093/bioinformatics/btag567 (PMC13503031; doi:10.1093/bioinformatics/btag567)
Supplement: Supplementary file 1 [file btag567_supplementary_data.zip › supplementary_figures.docx]

**Supplementary Data**

**Figure S1:** **Schematic overview of the Snakemake workflow.** Pairwise comparisons are performed between all input genomes. For each genome pair, FASTA and GFF3 files are processed to assess chromosome number consistency. When genomes share the same number of chromosomes, whole-genome alignment is conducted using *nucmer* or *minimap2*, followed by structural variant (SV) detection with SyRI, producing BEDPE output for visualization in SynFlow SV graphs. In parallel, protein sequences are extracted (*gffread*), indexed (*DIAMOND makedb*), and aligned (*DIAMOND blastp*). Reciprocal best hits are identified (*bbmh.py*), followed by collinearity detection (*MCScanX*) and anchor generation (*colinearity2anchor.py*), resulting in inputs for SynFlow collinearity graphs. For genome pairs with differing chromosome numbers, collinearity-derived anchors are converted to BEDPE format (*colinearity2bepe.py*) to enable SV-like visualization**.**

**Figure S2:** **Schematic representation of structural variation and synteny patterns visualized in SynFlow.** Illustrative examples of genomic relationships between two sequences (top: reference; bottom: query) are shown. Conserved syntenic regions are depicted as collinear connections (grey). Structural rearrangements include inversions (orange), translocations (green), inverted translocations (green with reversed orientation), and reciprocal translocations. Duplication is represented by duplications (blue), while insertions correspond to sequence present in one genome but absent in the other. Complex or nested events combine multiple rearrangement types within the same region. Colored links indicate the type and orientation of genomic rearrangements between corresponding loci.

**Figure S3:** **Comparative visualization of structural variation and synteny in Musa genomes using SynFlow.** (A) Published static synteny plots of banana (*Musa* spp.) assemblies showing large reciprocal translocations (from Martin et al, 2025). Colored ribbons connect homologous chromosome segments, with evolutionary relationships illustrated on the left. (B) Similar data re-rendered in SynFlow and colored in a similar way via the interface of the application. For comparison purposes, only the intensity of the color segments was adjusted in Inkscape, and the size of the chromosomes was adjusted manually.
